# A novel model to quantify balance alterations in older adults based on the center of pressure (CoP) measurements with a cross-sectional study

**DOI:** 10.1371/journal.pone.0256129

**Published:** 2021-08-16

**Authors:** Ángel Gabriel Estévez-Pedraza, Lorena Parra-Rodríguez, Rigoberto Martínez-Méndez, Otniel Portillo-Rodríguez, Zoraida Ronzón-Hernández

**Affiliations:** 1 School of Engineering, Universidad Autónoma del Estado de México, Toluca de Lerdo, Mexico; 2 Research Department, Instituto Nacional de Geriatría, Mexico City, Mexico; 3 Centre for Research in Social Sciences and Humanities, Universidad Autónoma del Estado de México, Toluca de Lerdo, Mexico; University of Alberta, CANADA

## Abstract

**Background:**

The timely detection of fall risk or balance impairment in older adults is transcendental because, based on a reliable diagnosis, clinical actions can be taken to prevent accidents. This study presents a statistical model to estimate the fall risk from the center of pressure (CoP) data.

**Methods:**

This study is a cross-sectional analysis from a cohort of community-dwelling older adults aged 60 and over living in Mexico City. CoP balance assessments were conducted in 414 older adults (72.2% females) with a mean age of 70.23 ± 6.68, using a modified and previously validated Wii Balance Board (MWBB) platform. From this information, 78 CoP indexes were calculated and analyzed. Multiple logistic regression models were fitted in order to estimate the relationship between balance alteration and the CoP indexes and other covariables.

**Results:**

The CoP velocity index in the Antero-Posterior direction with open eyes (MVELAPOE) had the best value of area under the curve (AUC) to identify a balance alteration (0.714), and in the adjusted model, AUC was increased to 0.827. Older adults with their mean velocity higher than 14.24 mm/s had more risk of presenting a balance alteration than those below this value (*OR* (*Odd Ratio*) = 2.94, *p*<0.001, 95% *C.I.*(*Confidence Interval*) 1.68–5.15). Individuals with increased age and BMI were more likely to present a balance alteration (*OR* 1.17, *p*<0.001, 95% *C.I.* 1.12–1.23; *OR* 1.17, *p*<0.001, 95% *C.I.* 1.10–1.25). Contrary to what is reported in the literature, sex was not associated with presenting a balance alteration (*p* = 0.441, 95% *C.I.* 0.70–2.27).

**Significance:**

The proposed model had a discriminatory capacity higher than those estimated by similar means and resources to this research and was implemented in an embedded standalone system which is low-cost, portable, and easy-to-use, ideal for non-laboratory environments. The authors recommend using this technology to support and complement the clinical tools to attend to the serious public health problem represented by falls in older adults.

## Introduction

Falls in older adults have always represented a serious global public health problem. The World Health Organization (WHO) estimates that 28–35% of people aged 65 and older experience falls each year. The percentage increases to 32–42% for those over 70 years [1]. Falls in older adults have significant consequences in individuals such as severe injuries, reduction in life expectancy, disabilities, fear of falling, complications with chronic diseases [2], or worse, death. Additionally, falls represent a high and significant cost for countries’ health systems and people [3].

Falls have a severe impact on senior citizens’ quality of life [4], which is why a risk analysis should be performed for them. Based on this analysis, clinical plans can be implemented (personally or guided by geriatric specialists) to prevent an accident or reduce the fall risk level. The balance assessment allows us to identify the risk level through characterizations based on clinical tools or technological resources.

For the clinical tools, about 30 instruments to assess fall risk and balance have been reported in systematic reviews and meta-analyses [5, 6]; however, it has also been documented that these tools have some disadvantages [7]. Firstly, the execution of demanding complex balance tasks, besides compromising a person’s physical integrity, leads to people not completing an evaluation [8]. Secondly, time consumption, stress, nervousness, and fatigue could result in the senior citizen refusing to collaborate [9]. Thirdly, subjectivity is caused by the tendency to rounded scores, ceiling effect, or the lack of knowledge and skills in managing geriatric conditions [10]. Additionally, the lack of coordination of the activities and roles because sometimes evaluators or evaluates do not understand each methodology. Finally, when patients go to private health institutions for evaluation, service fees can be potentially high or raise concerns about fraud and abuse [7].

The most common technique to evaluate the balance is the measurement and assessment of the center of pressure (CoP) trajectory, which represents the average of the absolute pressure exerted by the body to the ground [11]. The CoP allows the characterization of the body sway quantitatively using statokinesiograms (time-series matching of CoP trajectories) and indexes/metrics derived from it [12]. The most common technical tools to evaluate the balance are stabilometers such as the Biodex Balance System^TM^ SD (Biodex Medical Systems, USA) [13] and force platforms (which characterize the human body equilibrium based on the recording of the CoP’s trajectory) [14]. Unfortunately, these systems have significant disadvantages in price and portability, limiting their use to gait clinics and specialized laboratories. For example, the Biodex stabilometer weighs 89 kg and costs approximately USD 21,000.00. On the other hand, force platforms require an assessment area of at least 2.5 m^2^ and external devices to operate correctly (signal acquisition modules and use of a computer), increasing its cost by up to USD 15,500.00 for a minimal system.

The use of any of these options requires trained personnel due to the technical complexities and the multiple functions and operation modes. Besides, the restricted access to CoP raw data limits the analysis and development of new fall risk estimators that could be more accurate. Some alternatives to these commercial systems are devices based on inertial sensors, video/depth cameras, force sensors, laser sensing, wearables (smart wrist-worn or body-worn sensors), etc., [15]. These prototypes are still under investigation for fall risk assessment. Furthermore, many of them require complex electronic instrumentation and high processing that must be performed on a computer. Furthermore, these devices need to be personalized and adapted for each subject.

In this work a modified Wii Balance Board™ (WBB) from Nintendo^®^ was used. This device was presented and has been validated in a previous work [16]. The Wii Balance Board™ (WBB) from Nintendo^®^ [17] is a suitable alternative to force platforms as stated by other studies [18, 19] where the WBB has been validated, resulting in it performing comparably to laboratory-grade force platforms for static standing computerized posturography [18]. Furthermore, the WBB presents low inter-device variability [17]; even after years of use, these devices do not present significant alterations in their measurements; and the battery charge level does not affect the sensor data [19]. The only reported drawback for this device is the unstable sampling frequency, but in this work that problem was addressed by using a modified WBB to provide stable sampling frequency, as reported in [16].

In this work, and based on previous studies [20–22], it was hypothesized that one or more indexes derived from the CoP measurements using a WBB should be capable to discriminate between people with and without balance alterations that could lead to falls. If so, it would be possible to use the WBB to easily and quickly predict subjects with a higher risk of falling.

The main contribution of this work, besides the methodology and algorithm presented, is the fact that the Modified Wii Balance Board platform (MWBB) provides a low-cost, portable, and easy-to-use system, ideal for non-laboratory environments [16], capable of estimating the probability of presenting a balance alteration in older adults and linking these measurements with fall risk.

## Methodology

### Study population and design

This study is a cross-sectional analysis from a cohort of community-dwelling older adults aged 60 and over living in Mexico City. They were recruited through informative talks and brochures inviting them to participate in the cohort at the National Institute of Geriatrics (Instituto Nacional de Geriatría) in Mexico City from January to April 2019. Persons eligible to participate were those who were able to mobilize with or without assisting devices, with total or slight independence (Barthel index score ≥60, [23]) and no cognitive impairment or slight impairment (Mini-Mental State Examination, MMSE ≥24, [24]). Those who were institutionalized, who had any acute or chronic condition, with vestibular disorders causing instability, dependency, or severe cognitive impairment or dementia that in medical staff’s judgment could affect the ability to complete the physical performance tests, were excluded. The study was approved by the Institute’s Ethics and Research Committees and registered under the number DI_PI-008/2018. Written informed consent was obtained from each subject before enrollment in the study. All the information collected is stored on the secure server of the National Institute of Geriatrics and only collaborators can access it.

### Data acquisition

**Fig 1:**
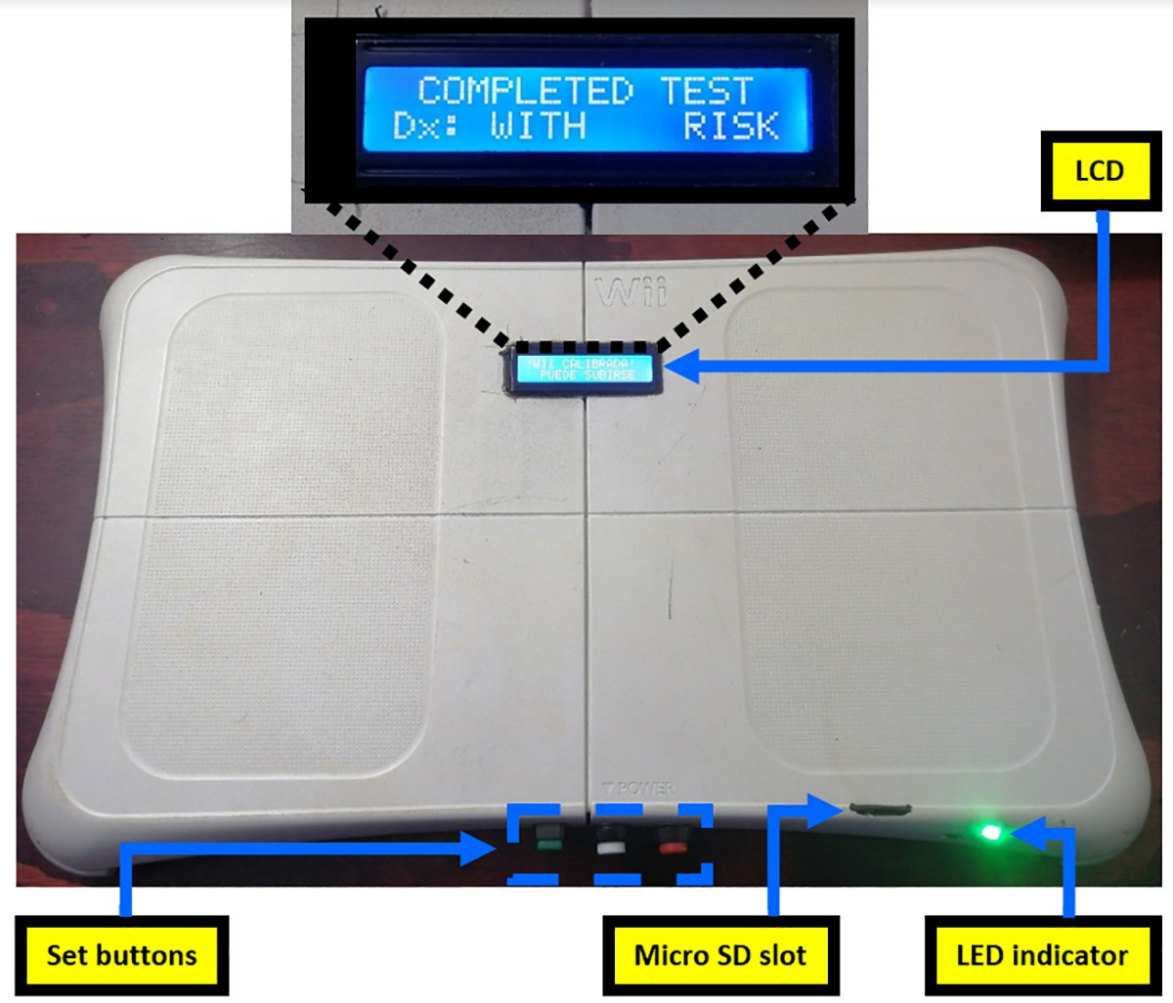
Modified Wii Balance Board (MWBB) to estimate a balance alteration.

A Modified Wii Balance Board platform (MWBB) was used in this study [16]. An embedded microcontroller records and processes the center of pressure (CoP) data at a stable sampling rate of 50 Hz. Subsequently, it computes metrics derived from the CoP signals (with a resolution of 1/100th of a millimeter). The device front can be seen in Fig 1. The only necessary condition to carry out a CoP measurement with this device is to hold an upright standing position for two minutes. The device interfaces (liquid crystal display (LCD), light-emitting diode (LED), and audibility) guide the evaluator during a test, making its operation simple and intuitive. The data obtained from the evaluations are recorded in labeled files (with date and time) and stored in a Micro SD memory. The three front buttons set the device’s built-in real-time clock (like an alarm clock configuration). More details and technical information can be found in [16].

### Test protocol and measurements

All evaluations were carried out at the Instituto Nacional de Geriatría facilities in the laboratory of research and functional evaluation of older adults (Laboratorio de Investigación y Evaluación Funcional del Adulto Mayor “LIEFAM”) by specialized staff in geriatrics. The total estimated time for the complete test battery was 2.5 hours on average per participant. After each participant read and agreed to sign the informed consent, their identification form was filled out. Data and measurements obtained included the following:

General characteristics: Data regarding age, sex, number of medications being taken, comorbidities (chronic diseases diagnosed by a physician) categorized by Charlson Comorbidity Index [25], and a fall questionnaire of the CDC [26] were obtained by self-reporting.

Assessment of vital signs: It included measurements of sitting and standing blood pressure, heart rate, respiratory rate, and blood glucose.

Assessment of physical performance: It included gait speed, leg strength, and balance assessments. Gait speed was recorded from a 6-meter usual pace walk (with the instruction to walk as if she/he were going to move from one room to another in her/his home) over an instrumented walkway GAITRite^®^ PLATINUM (GAITRite, USA), at a sampling rate of 100 Hz. Leg strength was evaluated by the number of sit-to-stand movements (squats in a chair without armrests) that each participant can complete in 30 seconds. Each subject performed the 4-Stage Balance Test using a Balance System SD^TM^ stabilometer to assess their static balance, asking each individual to perform parallel, semi-tandem, tandem, and one-legged stance.

Anthropometric measurements: Feet length, feet width, legs length, weight, and height were measured and recorded.

Balance tests with the MWBB: The test protocol for the balance measurement with the MWBB is based on the Romberg test to assess human equilibrium [27]. It consists of placing the subjects on the platform surface with their feet together (very closely positioned, side by side, and no opening angle), barefoot, assuming the most upright posture possible, with arms crossed over the chest [28]. During the evaluation, individuals focused their attention on a fixed point in front, adjusted at eye level, and located half a meter apart in distance.

**Fig 2:**
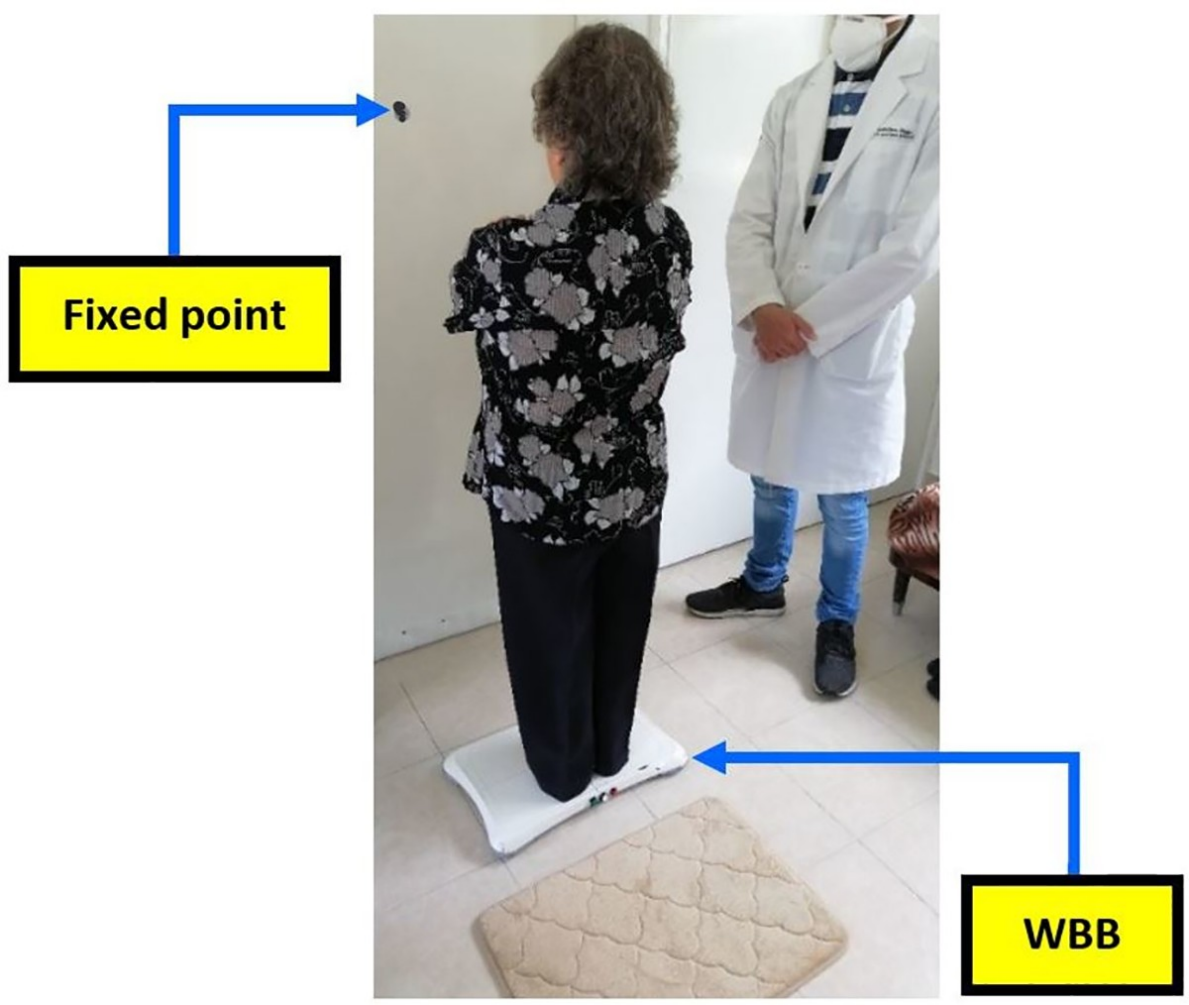
Center of pressure (CoP) balance assessment with the Modified Wii Balance Board (MWBB).

Once the participants stand on the MWBB, the device detects their presence (programmed threshold of 40 kg) and starts a 5-second countdown, which indicates the start of a test. Once this countdown is finished, the device automatically records the CoP data for one minute, and immediately after, through an auditory stimulus, the subjects are instructed to close their eyes, recording another minute. This sequential process ensured that the person’s feet remained in the same position on the platform surface in both visual conditions. From the recorded CoP signals, 78 CoP indexes in the time (distance, area, and hybrid) and frequency domain are generated by the MWBB (39 with eyes open and 39 with eyes closed). The description and formulas of these metrics can be found in [11]. The front LED in green indicates correct data storage and indexes calculation at the end of the test. Fig 2 shows a subject during a CoP measurement with the MWBB.

### Variables

The primary endpoint considered was balance alteration, defined when individuals could not hold the feet-together, semi-tandem, and tandem positions for ten seconds without moving their feet or needing support, or when participants could not maintain the one-legged stance for five seconds.

The 78 CoP metrics provided by the MWBB were used as continuous variables, as well as age, body mass index (BMI), gait speed, and the number of squats performed. On the other hand, sex (man or woman), falls in the previous year (yes, no), multimorbidity (Charlson comorbidity index equal to or greater than three), and polypharmacy (five or more medications consumed daily) were considered as dichotomic variables.

### Statistical analysis

The sample size was calculated using the Cochran’s formula for large populations as indicated below:

$$n=\frac{z^{2}pq}{d^{2}}$$

Where:

- *z* = 1.96, assuming a 95% confidence level.
- *p* = estimated proportion of population that experience falls in Mexico, in this case 40.4% [29].
- *q* = 1−*p*.
- *d* = is the desired level of precision, in this case 5%.

This formula resulted in 370 participants needing to achieve this confidence level. Nevertheless, a total of 414 participants were included.

A descriptive analysis was performed, where continuous variables were represented using means and standard deviations, and categorical variables were expressed as numbers and percentages. The normality of the continuous variables was assessed using Shapiro-Wilk and Kolmogorov-Smirnov tests. Comparisons of individuals with and without balance alterations were estimated through a t-test for parametric variables, a Mann-Whitney test for non-parametric variables, and a χ^2^ test for categorical variables. The predictive validity of a balance alteration for the 78 CoP indexes was assessed using the Hosmer-Lemeshow Goodness of Fit test and the area under the receiver-operating characteristic curve (AUC). The optimal cut-off point was obtained for the index with the higher AUC that best distinguishes between people with and without a balance alteration, based on Youden’s statistic. Finally, a multiple logistic regression model was fitted to estimate the association between presenting a balance alteration and the rest of the covariables. The final model was adjusted for sex, age, and BMI. All the analyses were processed using the Stata^®^ Statistical Software (version 15.1, StataCorp, USA).

## Results

A total of 497 individuals were included in the cohort; 83 participants were excluded for various reasons (30 individuals were under the age of 60, 53 did not complete the test, got off the platform, or held onto the guardrail, impeding the CoP measurements from being carried out). This resulted in a total of 414 participants with a mean age of 70.23 ± 6.68. From this total, 72.2% (n = 299) were women and 27.8% (n = 115) were men. From the total of subjects 39.4% reported having suffered a fall in the previous year, 28.7% scored three or more in the Charlson comorbidity index, and 40.6% took five or more medications daily (Table 1). It can be observed from Table 1 that the individuals presenting a balance alteration are older, have a higher prevalence of comorbidity, take more medications, have a greater BMI, and perform poorly in the physical performance tests for gait speed and the number of squats. It is interesting to note that the variables of sex and previous falls did not present statistically significant differences.

**Table 1:** General characteristics by balance alteration.

	Total	Without balance alteration	With balance alteration	p value
	n = 414	n = 299	n = 115	
Age, (years)	70.23 ± 6.68	68.49 ± 6.07	74.73 ± 6.10	<0.001
Sex, women, n (%)	299 (72.2)	216 (72.2)	83 (72.2)	0.989
Multimorbidity, n (%)	119 (28.7)	71 (23.7)	48 (41.7)	<0.001
Polypharmacy, n (%)	168 (40.6)	105 (35.1)	63 (54.8)	<0.001
Reported falls in the last year, n (%)	163 (39.4)	116 (38.8)	47 (40.9)	0.699
BMI, (kg/m^2^)	27.56 ± 4.31	26.95 ± 3.92	29.15 ± 4.86	<0.001
Gait speed, (m/s)	103.98 ± 25.00	108.22 ± 23.73	92.93 ± 24.95	<0.001
Total of squats	9.91 ± 2.83	10.35 ± 2.88	8.77 ± 2.88	<0.001

In the measurements with the MWBB, statistically significant differences were found between individuals with and without balance alteration in 57 of the 78 CoP indexes (the complete list with this information is shown in S1 Table). Table 2 shows the 10 CoP indexes with the highest AUC resulting from the ROC analysis (all with a statistical difference in balance alteration). It is interesting to note that half of these belong to the time domain and 5 to the frequency domain. The highest AUC was found for the mean velocity in the antero-posterior (AP) displacement with eyes open, MVELAPOE (AUC = 0.714, sensitivity = 0.496, specificity = 0.836, positive predictive value = 0.538, negative predictive value = 0.812). Following the Youden index analysis, the optimal cut-off value of MVELAPOE, providing the best trade-off between sensitivity and specificity for identifying a balance alteration, was 14.24 mm/s. From this value, the mean velocity in AP displacement with eyes open was dichotomized (MVELAPOE_DIC), indicating a balance problem if a person evaluated with the MWBB obtains a result equal to or greater than 14.24 mm/s in the continuous variable.

**Table 2:** Center of pressure (CoP) indexes with the highest area under the curve (AUC) related to balance alteration and descriptive analysis for the dichotomized mean velocity in the antero-posterior displacement with eyes open (MVELAPOE_DIC).

		Total	Without Balance Alteration	With Balance Alteration	p value	AUC (95% C.I.)
		n = 414	n = 299	n = 115		
MVELAPOE	[mm/s]	12.12 ± 5.65	10.88 ± 4.43	15.35 ± 7.06	<0.001	0.714 (0.658–0.770)
POWERAPOE		16.60 ± 16.89	14.42 ± 14.74	22.26 ± 20.49	<0.001	0.676 (0.619–0.733)
MVELOE	[mm/s]	19.25 ± 7.87	17.75 ± 6.38	23.15 ± 9.85	<0.001	0.674 (0.614–0.733)
MFREQAPOE	[Hz]	0.48 ± 0.17	0.45 ± 0.16	0.54 ± 0.18	<0.001	0.656 (0.598–0.714)
AREASWOE	[mm^2^/s]	47.74 ± 36.77	41.92 ± 29.67	62.86 ± 47.72	<0.001	0.652 (0.590–0.713)
CFREQAPOE	[Hz]	0.69 ± 0.16	0.67 ± 0.16	0.75 ± 0.16	<0.001	0.642 (0.585–0.698)
POWER95APOE	[Hz]	1.36 ± 0.36	1.31 ± 0.36	1.48 ± 0.35	<0.001	0.642 (0.584–0.699)
MVELAPCE	[mm/s]	18.83 ± 10.79	17.43 ± 9.69	22.47 ± 12.56	<0.001	0.635 (0.576–0.694)
POWER50APOE	[Hz]	0.38 ± 0.10	0.37 ± 0.10	0.40 ± 0.10	<0.001	0.627 (0.569–0.686)
POWERRDOE		10.96 ± 9.56	9.86 ± 8.43	13.82 ± 11.58	<0.001	0.626 (0.564–0.688)
MVELAPOE_DIC* (MVELAPOE<14.24 mm/s)	n (%)	106 (25.60)	49 (16.38)	57 (49.56)	<0.001	0.667 (0.615–0.716)

* Chi Square test was made for MVELAPOE_DIC for group comparisons.

**Fig 3:**
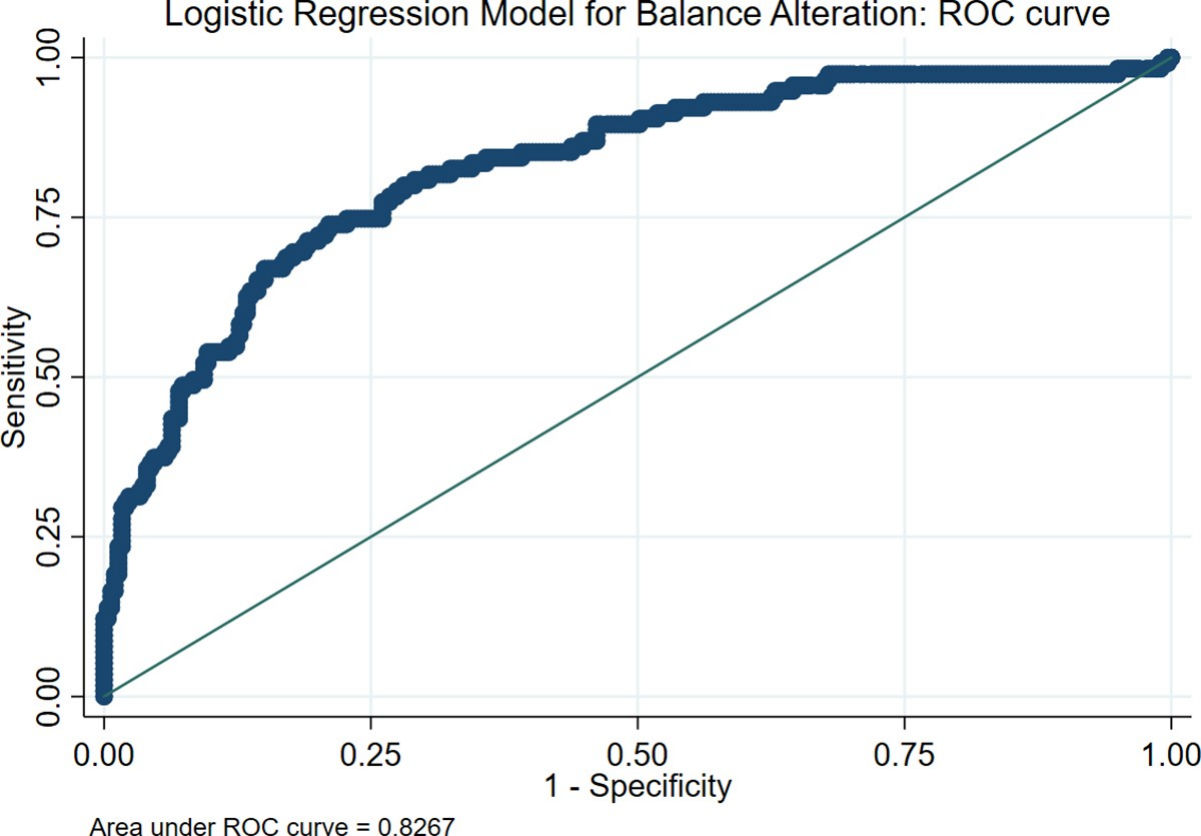
Logistic regression model for balance alteration. ROC curve. ROC curve between the logistic regression model (fitted by the dichotomized mean velocity in antero-posterior displacement with eyes open, sex, age, and BMI) with balance alteration.

The full logistic regression model for balance alteration containing three covariates (Table 3) was statistically significant (n = 414; goodness-of-fit test χ^2^ = 7.147, d.f. = 8, p = 0.521; area under the ROC curve = 0.827). Individuals with MVELAPOE higher than 14.24 mm/s were 2.94 times more likely to exhibit a balance alteration than individuals with MVELAPOE below 14.24 mm/s. Increasing age (OR 1.17, p<0.001) and BMI (OR 1.17, p<0.001) were associated with an increased likelihood of presenting a balance alteration, but contrary to what is reported in the literature [30], in this model, sex was not related to balance alteration. Fig 3 shows the graph of the area under the curve of the fitted model.

**Table 3:** Logistic model to estimate balance alteration.

Variable	OR	β	p value	95% C.I.	
MVELAPOE_DIC	2.94	1.08	<0.001	1.68	5.15
Sex (women)	1.26	0.23	0.441	0.70	2.27
Age, years	1.17	0.16	<0.001	1.12	1.23
BMI, kg/m^2^	1.17	0.16	<0.001	1.10	1.25
Constant	<0.001	-17.32	<0.001	<0.001	<0.001
		Hosmer-Lemeshow Goodness-of-fit test, p-value:			0.339
			Area under ROC curve:		0.827

The model has a sensitivity of 0.478, a specificity of 0.930, a positive predictive value of 0.724, a negative predictive value of 0.826, and correctly predicts a possible balance alteration 82.7% of the time. In other words, it can provide a reliable diagnosis of a balance problem in 4 out of 5 people, based on a one-minute CoP balance assessment since only the results for open eyes are needed, halving the evaluation time, and optimizing the computational resources of the MWBB. Finally, it is possible to compute the probability of presenting a balance alteration (*P_BA_*) using Eq 1.


$$P_{BA}=\frac{1}{1+e^{-(\beta_{0}+\beta_{1}X_{1}+\beta_{2}X_{2}+\beta_{3}X_{3}+\beta_{4}X_{4})}}$$ (1)

Where the *β*_*i*_ are the regression coefficients (given in Table 3), and the *X*_*i*_ are the regression model variables with *i =* 0, 1, 2, 3, and 4: the constant of the regression, MVELAPOE_DIC, sex, age, and BMI, respectively. For example, a 70-year-old male participant with a MVELAPOE below 14.24 mm/s, and a BMI = 22.70 kg/m2, has a 7.17% probability of presenting a balance alteration. The probability of presenting a balance alteration for an 80-year-old female participant, with a MVELAPOE above 14.24 mm/s, and a BMI = 33.20 kg/m^2^ is 88.28%.

## Discussion

In this study, an association between presenting a balance alteration and the mean velocity of the CoP in the antero-posterior direction with open eyes (MVELAPOE) was found using a modified WBB. A cut-off point of 14.24 mm/s was established for MVELAPOE (AUC ROC of 0.827) to maximize the model’s specificity and sensitivity. Older adults with their mean velocity higher than this cut-off point present more balance alterations than those below this value. Moreover, when adjusted by sex, age, and BMI, these individuals have almost three times the odds of presenting a balance alteration.

Various factors influence balance and mobility in older people. Several studies have investigated the influence of age, sex, and BMI on balance and falls. In general, age is significantly correlated with balance [31]. Studies also revealed sex differences in balance performance [32, 33]. On the other hand, some studies have suggested that the BMI should be used as an initial step in the determination of health risks, and many have investigated its relationship with balance. Some have shown that BMI did not influence balance [34], but others reported increased risk of falling associated with obesity [35]. For these reasons, we included these covariables in the final model. Even though sex was not significatively associated, we decided to keep it due to its biological relevance.

From the results, it can be established that of the 78 original CoP indexes analyzed, 73% were able to identify a balance problem, with MVELAPOE being the best at discriminating between groups. Rocchi et.al. [36, 37] reported that this parameter is part of the minimum optimal set of CoP variables describing postural sway in people with Parkinson’s disease (along with RMS value, mean distance, ranges, area of 95% confidence circle, area of 95% confidence ellipse, median frequency, 95% power frequency, centroidal frequency, and frequency dispersion). However, further research is needed to select indexes with high specificity and reliability in intergroup classifications. These results indicate that the indexes commonly studied for CoP signals provide different information depending on the origin of the equilibrium alterations.

It is possible to compare the performance of our model with other studies that use CoP stabilometry to assess balance and fall risk, despite high variability between methodological variables, such as time of day, populations, sample sizes, outcomes, instrumentation, and analysis parameters [38]. The first three papers in Table 4 used a WBB as a CoP measuring device, and the subsequent four studies used a force platform as the sensing system. The main difference between our study and the others is the better AUC value obtained (0.827) and the specificity obtained (0.930), regardless of data processing, even though a force platform is considered the gold standard in CoP measurement for characterizing the human equilibrium [39]. Only the work of Hsieh [40] reports slightly better results in AUC (0.837), but their methodology included a force platform, a smartphone, and an external computer, which represents an advantage for our approach that only depends on a standalone device.

**Table 4:** Some works about fall risk estimation, fall prediction, balance impairment, and fall identification in older adults.

Author/Year	Device/Feet position	Methodology	Sample size	Assessment time	Metrics and performance
This work.	A modified WBB.	ROC curve & multivariable logistic regression.	414	1 min.	• CoP velocity AP OE: AUC = 0.827. SE = 0.478, SP = 0.930.
[20] /2015.	WBB & computer /comfortable stance.	ROC curve & multivariable logistic regression.	73	1 min.	• CoP velocity AP OE: AUC = 0.67. • CoP velocity ML OE: AUC = 0.71. Sensitivity & Specificity: NR
[21] /2016.	WBB & computer /comfortable stance.	ROC curve & Ranking Forest Algorithm.	84	1 min.	• Composed Index RFK: AUC = 0.75. Sensitivity & Specificity: NR
[22] /2017.	Two WBB & computer /comfortable stance.	ROC curve & discriminant functions.	100	1 min.	• CoP velocity AP OC: AUC = 0.688. SE = 0.833, SP = 0.447. • CoP velocity OC: AUC = 0.691. SE = 0.833, SP = 0.426. • RQ CoP velocity AP: AUC = 0.660. SE = 0.833, SP = 0.404. • RQ CoP velocity: AUC = 0.670. SE = 0.833, SP = 0.404.
[41] /2016.	Force platform & computer / 6-inch feet separation.	ROC curve & logistic regression.	75	2 min.	• 95% Conf Ellipse CE: AUC = 0.574. • CoP velocity CE: AUC = 0.584. • CoP SD ML CE: AUC = 0.612. • CoP SD AP CE: AUC = 0.591. • Shannon Entropy: AUC = 0.539. • Renyi Entropy: AUC = 0.596. Sensitivity & Specificity: NR
[42]/2018.	Force platform & computer / One-legged stance.	ROC curve.	170	2.5 min.	• CoP velocity ML OE: AUC = 0.65. SE = 0.70, SP = 0.58. • CoP velocity AP OE: AUC = 0.68. SE = 0.78, SP = 0.54. • 95% Conf Ellipse OE: AUC = 0.72. SE = 0.66, SP = 0.68.
[43] /2019.	Force platform & computer / 10 cm feet separation and 20^o^ opening angle and comfortable stance.	Multi-Layer Perceptron (MLP), Support Vector Machines (SVM), Naïve Bayes (NB), K-Nearest Neighbours (KNN) & ROC curve.	73	12 min.	10 time-domain CoP measures and 5 frequency-domain CoP measures: • MLP mean metric: AUC = 0.77. • SVM mean metric: AUC = 0.71. • KNN mean metric: AUC = 0.70. • NB mean metric: AUC = 0.73. Sensitivity & Specificity: NR
[40] /2019.	Force platform, smartphone & computer / comfortable stance.	ROC curve	30	7 min.	• CoP velocity ML OE: AUC = 0.761. • CoP velocity AP OE: AUC = 0.698. • 95% Conf Ellipse CE: AUC = 0.788. • RMS Acceleration AP by smartphone OE: AUC = 0.761. • RMS Acceleration AP by smartphone OC: AUC = 0.837. Sensitivity & Specificity: NR

SE: Sensibility; SP: Specificity; NR: Not Reported.

Another essential contribution of our approach is the sample size. Some of the authors in Table 4 state that their reduced sample size could compromise and limit their research conclusions. The sample recruited for our study was carefully designed, considering data on the significance level and the proportion of the studied population.

The assessment time is another crucial variable that could substantially influence the research results. In Table 4, the test’s duration from 1 to 12 minutes (composed of multiple trials) is reported. According to the International Society for Posture and Gait Research, when assessing older adults it is necessary to keep the assessment time as short as possible to minimize tiredness and fall risk [44]. The 53 individuals that were excluded from our analysis could not complete the test when they had to close their eyes. Our assessment time is cut by half and reduced to just one minute since MVELAPOE is a metric obtained from an eyes-open measurement, helping maintain the participants’ health and well-being.

Finally, it is worthwhile to note that the equipment required by the other studies is more complex and expensive than our modified WBB. Even those studies that used a WBB required an external computer to obtain and process the data, increasing the cost and complexity of the system. Our proposed device [16] performs the data acquisition and processing within 10 seconds after evaluation without needing more components. As a simple embedded standalone device like a bath scale, it can have a relevant impact on clinical practice and research.

This study has some limitations: The main limitation is that we did not find an association between falls reported in the previous year and balance alteration, which could be because the studied population was independent, active, and without any acute conditions, so a broader sample is needed to verify these results. These characteristics of the sample could also impact the sensitivity of the final model, and more research is needed to improve this value. Finally, inter-rater and test-retest reliability and predictive validity tests are required to validate the complete sensing system and the model’s precision and effectiveness.

## Conclusions

Identifying older adults with balance alterations is a paramount public health concern that requires a multidisciplinary approach. Attending the complex multifactorial nature of the fall risk assessments, the technology can provide useful tools to support and complement diagnostics provided by clinical evaluations.

Using a low-cost device to acquire CoP displacements it was possible to create a statistical model to discriminate between people with and without balance alterations. As it is known, balance alterations are correlated with fall of risk, so, using our proposed model and a WBB it is possible to infer risk of fall in an easy and quick test.

According to our results, only one index derived from the CoP and data such as age, sex, and BMI are enough to determine if a subject suffers from balance alterations.

Our proposed modified WBB has the potential to provide a low-cost, portable, and easy-to-use system, ideal for helping in the timely identification of older adults with balance alterations that could lead to falls. However, more research is needed to validate the system as a medical tool and ensure the results’ reliability.

## Supporting information

S1 Table Complete list of the center of pressure (CoP) indexes performance respect to balance alteration. (DOCX)

S1 Data (CSV)
