# Supplementary material for: A novel model to quantify balance alterations in older adults based on the center of pressure (CoP) measurements with a cross-sectional study
Source: PLoS One. 2021 Aug 16;16(8):e0256129. doi: 10.1371/journal.pone.0256129 (PMC8366986; doi:10.1371/journal.pone.0256129)
Supplement: S1 Table — (DOCX) [file pone.0256129.s001.docx]

S1 Table. Full list of CoP indexes performance respect to balance alteration.

|  |  | Total | Without Balance Alteration | With Balance Alteration | p value | AUC (95% C.I.) |
| --- | --- | --- | --- | --- | --- | --- |
|  |  | n = 414 | n = 299 | n = 115 |  |  |
| MDISTOE | [mm] | 6.91 ± 2.23 | 6.65 ± 2.11 | 7.58 ± 2.42 | <0.001 | 0.621 (0.559-0.684) |
| MDISTCE | [mm] | 8.20 ± 2.86 | 7.87 ± 2.58 | 9.04 ± 3.34 | 0.002 | 0.599 (0.536-0.663) |
| MDISTMLOE | [mm] | 4.16 ± 1.41 | 4.01 ± 1.31 | 4.56 ± 1.57 | 0.001 | 0.608 (0.545-0.672) |
| MDISTMLCE | [mm] | 5.26 ± 2.04 | 5.05 ± 1.85 | 5.79 ± 2.41 | 0.009 | 0.583 (0.520-0.647) |
| MDISTAPOE | [mm] | 4.62 ± 1.68 | 4.44 ± 1.62 | 5.08 ± 1.75 | <0.001 | 0.617 (0.556-0.678) |
| MDISTAPCE | [mm] | 5.17 ± 1.82 | 4.95 ± 1.68 | 5.72 ± 2.05 | <0.001 | 0.614 (0.553-0.676) |
| RDISTOE | [mm] | 7.96 ± 2.63 | 7.66 ± 2.46 | 8.74 ± 2.88 | <0.001 | 0.620 (0.558-0.682) |
| RDISTCE | [mm] | 9.46 ± 3.39 | 9.07 ± 3.03 | 10.48 ± 4.01 | 0.002 | 0.600 (0.537-0.663) |
| RDISTMLOE | [mm] | 5.31 ± 1.80 | 5.12 ± 1.67 | 5.81 ± 2.03 | 0.001 | 0.604 (0.541-0.667) |
| RDISTMLCE | [mm] | 6.74 ± 2.69 | 6.47 ± 2.43 | 7.44 ± 3.18 | 0.008 | 0.585 (0.521-0.648) |
| RDISTAPOE | [mm] | 5.85 ± 2.14 | 5.62 ± 2.05 | 6.45 ± 2.26 | <0.001 | 0.617 (0.556-0.678) |
| RDISTAPCE | [mm] | 6.55 ± 2.33 | 6.27 ± 2.11 | 7.30 ± 2.70 | <0.001 | 0.614 (0.552-0.676) |
| TOTEXOE | [mm] | 923.99 ± 377.91 | 852.05 ± 306.22 | 1111.05 ± 472.65 | <0.001 | 0.674 (0.614-0.733) |
| TOTEXCE | [mm] | 1446.33 ± 761.90 | 1375.65 ± 708.48 | 1630.08 ± 862.39 | 0.003 | 0.595 (0.533-0.656) |
| TOTEXMLOE | [mm] | 589.25 ± 231.67 | 557.76 ± 200.25 | 671.12 ± 283.29 | <0.001 | 0.613 (0.549-0.678) |
| TOTEXMLCE | [mm] | 928.71 ± 488.04 | 906.87 ± 470.39 | 985.48 ± 529.16 | 0.156 | 0.545 (0.482-0.608) |
| TOTEXAPOE | [mm] | 581.85 ± 271.33 | 522.25 ± 212.70 | 736.82 ± 338.98 | <0.001 | 0.714 (0.658-0.770) |
| TOTEXAPCE | [mm] | 903.76 ± 517.94 | 836.44 ± 465.26 | 1078.78 ± 602.68 | <0.001 | 0.635 (0.576-0.694) |
| MVELOE | [mm/s] | 19.25 ± 7.87 | 17.75 ± 6.38 | 23.15 ± 9.85 | <0.001 | 0.674 (0.614-0.733) |
| MVELCE | [mm/s] | 30.13 ± 15.87 | 28.66 ± 14.76 | 33.96 ± 17.97 | 0.003 | 0.595 (0.533-0.656) |
| MVELMLOE | [mm/s] | 12.28 ± 4.83 | 11.62 ± 4.17 | 13.98 ± 5.90 | <0.001 | 0.613 (0.549-0.678) |
| MVELMLCE | [mm/s] | 19.35 ± 10.17 | 18.89 ± 9.80 | 20.53 ± 11.02 | 0.156 | 0.545 (0.482-0.608) |
| MVELAPOE | [mm/s] | 12.12 ± 5.65 | 10.88 ± 4.43 | 15.35 ± 7.06 | <0.001 | 0.714 (0.658-0.770) |
| MVELAPCE | [mm/s] | 18.83 ± 10.79 | 17.43 ± 9.69 | 22.47 ± 12.56 | <0.001 | 0.635 (0.576-0.694) |
| sRDOE |  | 3.93 ± 1.46 | 3.78 ± 1.35 | 4.32 ± 1.64 | <0.001 | 0.612 (0.550-0.673) |
| sRDCE |  | 4.69 ± 1.91 | 4.49 ± 1.66 | 5.23 ± 2.37 | 0.003 | 0.594 (0.531-0.656) |
| AREACCOE | [mm^2^] | 626.59 ± 489.01 | 576.86 ± 444.04 | 755.88 ± 572.34 | <0.001 | 0.618 (0.556-0.680) |
| AREACCCE | [mm^2^] | 903.20 ± 771.01 | 815.38 ± 642.92 | 1131.52 ± 1000.13 | 0.002 | 0.599 (0.536-0.661) |
| SAPMLOE |  | -2.52 ± 10.59 | -2.48 ± 9.14 | -2.62 ± 13.70 | 0.876 | 0.495 (0.431-0.559) |
| SAPMLCE |  | -0.52 ± 13.94 | -0.03 ± 11.16 | -1.78 ± 19.39 | 0.115 | 0.450 (0.385-0.515) |
| AREACEOE | [mm^2^] | 621.84 ± 458.95 | 572.07 ± 421.66 | 751.23 ± 524.36 | <0.001 | 0.623 (0.561-0.685) |
| AREACECE | [mm^2^] | 904.86 ± 725.39 | 819.82 ± 619.64 | 1125.98 ± 913.43 | 0.001 | 0.602 (0.539-0.665) |
| AREASWOE | [mm^2^/s] | 47.74 ± 36.77 | 41.92 ± 29.67 | 62.86 ± 47.72 | <0.001 | 0.652 (0.590-0.713) |
| AREASWCE | [mm^2^/s] | 92.18 ± 91.51 | 83.00 ± 78.70 | 116.05 ± 115.56 | 0.003 | 0.595 (0.533-0.657) |
| MFREQOE | [Hz] | 0.45 ± 0.12 | 0.43 ± 0.12 | 0.49 ± 0.13 | <0.001 | 0.618 (0.557-0.678) |
| MFREQMLOE | [Hz] | 0.53 ± 0.14 | 0.52 ± 0.14 | 0.54 ± 0.14 | 0.110 | 0.551 (0.487-0.614) |
| MFREQAPOE | [Hz] | 0.48 ± 0.17 | 0.45 ± 0.16 | 0.54 ± 0.18 | <0.001 | 0.656 (0.598-0.714) |
| MFREQCE | [Hz] | 0.58 ± 0.18 | 0.57 ± 0.18 | 0.59 ± 0.17 | 0.112 | 0.550 (0.488-0.613) |
| MFREQMLCE | [Hz] | 0.64 ± 0.19 | 0.65 ± 0.19 | 0.63 ± 0.17 | 0.730 | 0.489 (0.427-0.551) |
| MFREQAPCE | [Hz] | 0.64 ± 0.24 | 0.62 ± 0.24 | 0.69 ± 0.24 | 0.005 | 0.588 (0.527-0.650) |
| FDCCOE |  | 1.86 ± 0.30 | 1.84 ± 0.28 | 1.91 ± 0.33 | <0.001 | 0.621 (0.562-0.681) |
| FDCEOE |  | 1.86 ± 0.30 | 1.84 ± 0.28 | 1.91 ± 0.33 | <0.001 | 0.622 (0.562-0.683) |
| FDCCCE |  | 1.98 ± 0.35 | 1.97 ± 0.35 | 2.00 ± 0.37 | 0.109 | 0.551 (0.488-0.614) |
| FDCECE |  | 1.97 ± 0.35 | 1.97 ± 0.35 | 2.00 ± 0.37 | 0.112 | 0.550 (0.488-0.613) |
| RANGEMLOE | [mm] | 31.69 ± 12.62 | 30.54 ± 11.75 | 34.71 ± 14.25 | 0.003 | 0.595 (0.533-0.658) |
| RANGEAPOE | [mm] | 33.35 ± 13.96 | 31.64 ± 12.85 | 37.81 ± 15.71 | <0.001 | 0.625 (0.565-0.685) |
| RANGEMLCE | [mm] | 41.38 ± 19.64 | 39.64 ± 17.17 | 45.90 ± 24.45 | 0.020 | 0.574 (0.512-0.636) |
| RANGEAPCE | [mm] | 39.09 ± 16.21 | 36.68 ± 13.38 | 45.35 ± 20.71 | <0.001 | 0.621 (0.560-0.682) |
| POWERRDOE |  | 10.96 ± 9.56 | 9.86 ± 8.43 | 13.82 ± 11.58 | <0.001 | 0.626 (0.564-0.688) |
| POWERMLOE |  | 19.05 ± 17.52 | 16.82 ± 14.16 | 24.82 ± 23.28 | <0.001 | 0.611 (0.547-0.674) |
| POWERAPOE |  | 16.60 ± 16.89 | 14.42 ± 14.74 | 22.26 ± 20.49 | <0.001 | 0.676 (0.619-0.733) |
| POWERRDCE |  | 16.19 ± 14.18 | 14.78 ± 12.88 | 19.84 ± 16.61 | 0.004 | 0.590 (0.527-0.654) |
| POWERMLCE |  | 34.79 ± 38.27 | 32.45 ± 38.13 | 40.89 ± 38.12 | 0.040 | 0.565 (0.501-0.630) |
| POWERAPCE |  | 26.22 ± 24.41 | 23.30 ± 20.59 | 33.83 ± 31.13 | <0.001 | 0.618 (0.556-0.680) |
| POWER50RDOE | [Hz] | 0.48 ± 0.13 | 0.47 ± 0.12 | 0.50 ± 0.13 | 0.027 | 0.570 (0.510-0.631) |
| POWER50MLOE | [Hz] | 0.39 ± 0.10 | 0.38 ± 0.10 | 0.39 ± 0.10 | 0.764 | 0.510 (0.447-0.572) |
| POWER50APOE | [Hz] | 0.38 ± 0.10 | 0.37 ± 0.10 | 0.40 ± 0.10 | <0.001 | 0.627 (0.569-0.686) |
| POWER50RDCE | [Hz] | 0.59 ± 0.17 | 0.60 ± 0.17 | 0.59 ± 0.16 | 1.000 | 0.500 (0.439-0.561) |
| POWER50MLCE | [Hz] | 0.45 ± 0.15 | 0.46 ± 0.16 | 0.43 ± 0.14 | 0.064 | 0.441 (0.380-0.503) |
| POWER50APCE | [Hz] | 0.46 ± 0.13 | 0.46 ± 0.14 | 0.47 ± 0.12 | 0.059 | 0.560 (0.499-0.621) |
| POWER95RDOE | [Hz] | 1.72 ± 0.41 | 1.68 ± 0.41 | 1.81 ± 0.41 | 0.003 | 0.594 (0.533-0.656) |
| POWER95MLOE | [Hz] | 1.31 ± 0.27 | 1.31 ± 0.27 | 1.31 ± 0.28 | 0.995 | 0.500 (0.436-0.565) |
| POWER95APOE | [Hz] | 1.36 ± 0.36 | 1.31 ± 0.36 | 1.48 ± 0.35 | <0.001 | 0.642 (0.584-0.699) |
| POWER95RDCE | [Hz] | 2.04 ± 0.53 | 2.01 ± 0.53 | 2.10 ± 0.51 | 0.044 | 0.564 (0.501-0.627) |
| POWER95MLCE | [Hz] | 1.44 ± 0.33 | 1.45 ± 0.34 | 1.43 ± 0.31 | 0.883 | 0.505 (0.441-0.568) |
| POWER95APCE | [Hz] | 1.52 ± 0.47 | 1.48 ± 0.46 | 1.64 ± 0.46 | 0.001 | 0.609 (0.548-0.670) |
| CFREQRDOE | [Hz] | 0.86 ± 0.18 | 0.84 ± 0.18 | 0.90 ± 0.18 | 0.001 | 0.605 (0.544-0.665) |
| CFREQMLOE | [Hz] | 0.67 ± 0.13 | 0.67 ± 0.12 | 0.67 ± 0.13 | 0.688 | 0.513 (0.449-0.577) |
| CFREQAPOE | [Hz] | 0.69 ± 0.16 | 0.67 ± 0.16 | 0.75 ± 0.16 | <0.001 | 0.642 (0.585-0.698) |
| CFREQRDCE | [Hz] | 1.02 ± 0.24 | 1.01 ± 0.24 | 1.04 ± 0.22 | 0.063 | 0.559 (0.497-0.621) |
| CFREQMLCE | [Hz] | 0.76 ± 0.16 | 0.76 ± 0.16 | 0.75 ± 0.14 | 0.857 | 0.494 (0.431-0.557) |
| CFREQAPCE | [Hz] | 0.79 ± 0.20 | 0.77 ± 0.20 | 0.84 ± 0.20 | 0.002 | 0.600 (0.540-0.661) |
| FREQDRDOE | [Hz] | 0.64 ± 0.04 | 0.64 ± 0.04 | 0.64 ± 0.04 | 0.217 | 0.539 (0.475-0.603) |
| FREQDMLOE | [Hz] | 0.61 ± 0.05 | 0.61 ± 0.05 | 0.62 ± 0.05 | 0.736 | 0.511 (0.446-0.576) |
| FREQDAPOE | [Hz] | 0.64 ± 0.05 | 0.64 ± 0.05 | 0.65 ± 0.05 | 0.189 | 0.542 (0.478-0.605) |
| FREQDRDCE | [Hz] | 0.63 ± 0.04 | 0.62 ± 0.03 | 0.64 ± 0.04 | 0.008 | 0.584 (0.520-0.647) |
| FREQDMLCE | [Hz] | 0.60 ± 0.06 | 0.60 ± 0.05 | 0.61 ± 0.06 | 0.022 | 0.573 (0.510-0.636) |
| FREQDAPCE | [Hz] | 0.63 ± 0.05 | 0.62 ± 0.05 | 0.64 ± 0.06 | 0.002 | 0.597 (0.533-0.660) |
